# Addressing inequalities in the identification and management of perinatal mental health difficulties: The perspectives of minoritised women, healthcare practitioners and the voluntary sector

**DOI:** 10.3389/fgwh.2022.1028192

**Published:** 2022-12-22

**Authors:** Zoe Darwin, Sarah L. Blower, Chandani Nekitsing, Sarah Masefield, Rifat Razaq, Louise Padgett, Charlotte Endacott, Kathryn Willan, Josie Dickerson

**Affiliations:** ^1^ School of Human and Health Sciences, Department of Allied Health Professions, Sport and Exercise, University of Huddersfield, Huddersfield, United Kingdom; ^2^ Department of Health Sciences, Faculty of Sciences, University of York, York, United Kingdom; ^3^ Bradford Institute for Health Research, Bradford Teaching Hospitals NHS Foundation Trust, Bradford, United Kingdom

**Keywords:** perinatal mental health, inequalities, inequity, COVID-19, trauma-informed, mixed methods, system change

## Abstract

**Background:**

Perinatal mental health (PMH) difficulties affect approximately one in five birthing women. If not identified and managed appropriately, these PMH difficulties can carry impacts across generations, affecting mental health and relationship outcomes. There are known inequalities in identification and management across the healthcare pathway. Whilst barriers and facilitators have been identified there is a lack of clarity about how these relate to the avoidable and unfair inequalities experienced by various groups of women. Further research is required to understand how to address inequalities in PMH.

**Aim:**

To understand the key factors that enable and hinder access to PMH care for women from minoritised groups across the PMH care pathway, and how these have been affected by the COVID-19 pandemic.

**Methods:**

A sequential mixed-methods approach gathered views and experiences from stakeholders in one region in northern England. This included an online survey with 145 NHS healthcare practitioners and semi-structured interviews with 19 women from ethnic minority and/or socio-economically deprived backgrounds who had experienced PMH difficulties, and 12 key informants from the voluntary and community sector workforce. Quantitative data were analysed using descriptive statistics and framework analysis was applied to qualitative data.

**Findings:**

Barriers and facilitators were mapped using a socio-technical framework to understand the role of (i) processes, (ii) people (organised as women, practitioners and others), (iii) technology, and (iv) the system as a whole in deepening or alleviating inequalities. Influences that were identified as pertinent to inequalities in identification and management included provision of interpreters, digital exclusion, stigma, disempowerment, distrust of services, practitioner attitudes, data capture, representation in the workforce, narrow rules of engagement and partnership working. Stakeholder groups expressed that several barriers were further compounded by the COVID-19 pandemic.

**Discussion:**

The findings highlight the need for change at the system level to tackle inequalities across the PMH care pathway. Four inter-connected recommendations were developed to enable this systems change: building emotional safety between professionals and women; making PMH a part of core healthcare business; increasing cultural competency specific to PMH; and enhanced partnership working.

## 1. Introduction

Perinatal mental health (PMH) difficulties are common, affecting approximately one in five birthing women. Mental health difficulties spanning pregnancy and the first postnatal year can carry impacts across generations, through affecting mental health and relationship outcomes. It has therefore been argued that there is “no health without perinatal mental health” (1).

Rates are likely higher in low- and middle-income countries (2) and, within high-income countries, vulnerability is greatest amongst minoritised groups, including women from ethnic minority backgrounds (3, 4). Rates of PMH difficulties in trans and non-binary birthing people are unknown, although initial evidence suggested there may be increased vulnerability (5).

Differences exist between countries concerning visibility of PMH in policy and health systems. Increasingly, countries in the Global North have routine mental health assessment within universal services (i.e., primary care, maternity, child health) as part of their national clinical guidance and local guidelines. However, this alone does not ensure that needs will be identified or met. An equity-focused re-analysis of a systematic review on the identification and management of common mental health difficulties in the perinatal period (6) provides evidence of inequalities existing within PMH care pathways. By following the equity extension for guidance on reporting systematic reviews (PROGRESS-Plus) (7), this re-analysis found consistent evidence that identification and management are likely inequitable for ethnic minority women in the UK, both those who do and do not speak English; whereas there was less consistent evidence concerning other PROGRESS-Plus characteristics (e.g., age, parity and partnership status).

Barriers and facilitators to accessing PMH support have been the subject of various reviews (8–11), repeatedly identifying issues at multiple levels (e.g., individual, healthcare practitioner, organisational, sociocultural, structural). Whilst these reviews have sometimes acknowledged potential inequalities concerning aspects such as language barriers and cultural differences concerning stigma (10), equity-focused approaches have been under-explored. A recent exception was a systematic review (3) focused specifically on the experiences of ethnic minority women concerning PMH help-seeking and services in Europe identified aspects such as cultural influences, awareness and beliefs, across the 15 included studies (all from the UK).

Knowledge of the inequalities experienced by minoritised women in PMH are not fully understood, and evidence-based recommendations to tackle these inequalities are lacking. Further research is needed to clarify and better understand the inequalities experienced and the barriers to appropriate care for minoritised women in order to inform the design of strategies to tackle inequalities in the identification and management of PMH difficulties (6).

Responding to this gap and to regional concerns about inequalities in access, the authors have undertaken a programme of research to understand inequalities in identification and management of PMH difficulties and to develop recommendations for how to make PMH care equitable for everyone. The focus of this paper is to describe our primary research with stakeholders, where the aim was to understand the key factors that enable and hinder disclosure and identification of PMH difficulties in universal services (e.g., primary care, maternity, child health), and access, referral and take-up of targeted/specialist PMH services; with an emphasis on inequalities.

Shortly after the research was commissioned, the COVID-19 pandemic unfolded, accompanied by increased rates of psychological distress, including depression and anxiety symptoms in perinatal women (12). This reflects the role of psychosocial circumstances in vulnerability to PMH conditions and the importance of understanding context. Recognised stressors linked to the COVID-19 pandemic have included bereavement, threat of infection (for self, for baby), financial strain (through loss of income), social isolation, living in close confinement, restrictions concerning partners in maternity services, and missed pregnancy and parenthood experiences (13, 14). Moreover, parents' access to their usual coping mechanisms have been impacted by the pandemic, included through restrictions (15). Evidence gaps remain concerning how PMH inequalities may vary in this context.

The objectives were:

(i) To identify inequalities faced by women during the PMH period, observed by frontline NHS healthcare practitioners (HCPs) working in both universal and specialist services, and staff working in the voluntary and community sector (VCS). This included their perspectives on health inequalities identified in previous research and included an opportunity to self-identify other inequalities. We also explored how these inequalities have been affected by the COVID-19 pandemic.
(ii) To understand the perspectives of women (parents) from minoritised groups concerning the key factors that enable and hinder: disclosure/identification of PMH difficulties in universal services; and access/referral/take-up of targeted PMH services. This included consideration of challenges and opportunities relating to service access presented by the COVID-19 pandemic. We use the terms disclosure and identification in recognition that PMH difficulties may be apparent to a HCP through women volunteering (i.e. disclosing) their distress, or through a professional actively identifying distress (e.g. through the use of assessment tools (identification). We use the term women to refer to birthing parents, consistent with the policy and research evidence being quoted, and the sample included here; we recognise however that not all birthing parents are women, and that not all women who are parents have themselves been pregnant.
(iii) To synthesise the findings from the different stakeholder groups (i.e. HCPs, VCS staff, women) to develop recommendations for addressing existing inequalities in PMH care.

## 2. Materials and method

This research adopted a pragmatic stance and sequential mixed-methods design whereby we considered the most appropriate data collection method for each stakeholder group then integrated learning across groups. Views and experiences were gathered from various stakeholders in a region of Northern England. This included: (i) an online survey with NHS HCPs; (ii) semi-structured interviews with women from ethnic minority and/or socio-economically deprived backgrounds; and (iii) semi-structured interviews with key informants (KIs) from the VCS (e.g., service staff). The VCS comprises independent self-governing organisations that are usually run not-for-profit and include charities and community groups.

The study was approved by National Health Service (NHS) North West - Greater Manchester East Research Ethics Committee on 14th June 2021 (REC Reference: 21/NW/0158, IRAS: 293657).

### 2.1. Recruitment and sampling

#### 2.1.1. Healthcare practitioners

There are 15 organisations within our study region providing NHS services to women and families specifically during the perinatal period, 14 took part in the study (one organisation declined on the basis that they had no capacity). HCPs were invited to take part in the study *via* an email distributed by a lead contact at each organisation. The email contained brief information about the study's purpose and methods and a weblink to access the anonymous electronic survey. Researcher contact details were also given in case a potential participant had any queries prior to making the decision to take part. The first page of the survey comprised an information sheet and in order to proceed to the first set of questions HCPs were required to endorse a statement indicating their informed consent. HCPs were eligible to take part if they met the following criteria:

• Worked in the study region
• Were involved in the provision of universal or specialist healthcare to perinatal families
• Employed in a role from one of the following practitioner groups:
  ○ Midwives, Health Visitors and GPs in universal services
  ○ Practitioners within specialist PMH services, both inpatient (Mother and Baby Unit, MBU) and community
  ○ Specialist Midwives and Health Visitors working within universal services
  ○ Increasing Access to Psychological Therapies (IAPT) practitioners, i.e. mental health practitioners who deliver talking therapies

#### 2.1.2. Women

Women were recruited into the study *via* two pathways. Specialist PMH community teams sent study invitations and information in the mail to women known to their service and who met the eligibility criteria. The study was also advertised *via* relevant social media channels operated by our project partners and relevant VCS organisations. In both instances, women who were interested in taking part and hearing more about the study were invited to contact the research team directly by phone, text or email. Those who expressed an interest were provided with more detailed information and were given the opportunity to ask questions. If a potential participant agreed to proceed an interview date and time was set and informed consent was taken before the interview commenced.

Women were eligible to take part in the interviews if they met all of the following criteria:

• had been pregnant in the past 3 years
• gave birth in the study region
• experienced symptoms of PMH difficulties – regardless of whether they had received a diagnosis
• were either offered a specialist PMH service even if they did not access it or they felt that they needed support but did not get it at the time
• felt well enough to participate in an interview at the time of the study
• had at least one of the following characteristics that is associated with inequalities in PMH care: not White British, and/or lived in an area with high levels of deprivation (i.e. a geographical area identified as being in the highest quintile of socioeconomic deprivation nationally, or identifying themselves as living in an area that feels unsafe, living in poor housing, or finding it difficult to make ends meet)

Information was available in the most commonly spoken languages in the study setting (English, Urdu, Punjabi, Mirpuri, Pahari, Hungarian and Polish). All of the women interviewed received a £20 high street voucher to compensate for their time.

#### 2.1.3. Voluntary and community sector key informants

In order to identify potential KIs from the VCS, we undertook a mapping exercise to identify relevant organisations operating in our study area that provide emotional support to women during the perinatal period. Organisations were identified through a combination of study team contacts and networks, searching the internet and consultation with our project partners. A list of 35 organisations were identified, from which 20 organisations were prioritised and approached *via* email and a follow-up telephone call. Organisations that work specifically with groups at risk of health inequalities (e.g., ethnic minority, low socio-economic status) were targeted.

VCS KIs were provided with study information and given the opportunity to ask questions about the study. If they agreed to proceed an interview date and time was set and informed consent was taken by the researcher at the commencement of the interview.

### 2.2. Data collection

Due to social distancing restrictions at the time of the study arising from the COVID-19 pandemic, information was collected remotely - online, by telephone or using secure NHS approved technology for videocalls (e.g., Microsoft Teams).

#### 2.2.1. Survey of healthcare practitioners

The survey generated both quantitative and qualitative data through a mix of closed questions with simple yes-no or multiple response formats and open-ended questions with free text response formats. Questions gathered background information on the respondent and perceptions of inequalities in the PMH pathway, also exploring experiences of what works well and service improvement ideas. The survey contained conditional formatting to ensure questions were tailored according to the respondent's role in the perinatal pathway, e.g., practitioners working in universal services (e.g., GP, midwife, health visitor) were asked questions that focus on identification, disclosure and referral whereas those working in specialist services (e.g., community mental health team, psychotherapist) were asked questions that focus on referral, access (i.e., initial contact) and take-up (i.e., utilisation or engagement) of targeted services.

Some questions were tailored to ask specifically about six groups (identified in previous research literature and informed by PROGRESS-PLUS) indicated to be at risk of experiencing inequalities in the PMH pathway (1. Women who do not speak English, 2. Ethnic minority women who speak English, 3. Women experiencing individual and/or area-based socioeconomic deprivation, 4. Multiparous women (having borne more than one child), 5. Women who are not partnered, or are lone parenting, 6. Women with learning difficulties and 7. Women with low literacy). However, due to an error, low literacy was not included in the survey completed by respondents who self-identified as specialist practitioners. The survey was piloted with three practitioners and refined before being distributed. The survey took approximately 15 min to complete and was designed and distributed using Qualtrics software.

#### 2.2.2. Interviews with women

Semi-structured interviews gathered women's experiences of barriers and different steps of the PMH care pathway; for example, discussion of mental health in universal services (maternity, health visiting, primary care), any referrals made (e.g., to specialist PMH midwives or health visitors) and their outcomes, access of specialist services and other services in relation to their mental health (e.g., community mental health teams, IAPT, counselling). The interviews also explored how this may have been impacted by the COVID-19 pandemic when applicable (e.g., perinatal period overlapped with the pandemic). An interview schedule was developed with questions informed by previous literature and local PMH reports, and through collaboration between the multi-disciplinary research team and specialist PMH professionals (available as supplementary material 1).

Interviews lasted 45 min to an hour and were audio-recorded and transcribed. We had the capacity to offer interviews in different languages, however all participants chose to conduct their interviews in English. The researcher conducting the interviews (RR) had previous experience of sensitive-topic interviewing and was able to conduct interviews in English, Urdu and Pashto. The interviews were audio recorded and transcribed. The interview schedule was reviewed after the first few interviews and revised to address issues of phrasing and suggest additional probes or prompts to explore key topics.

#### 2.2.3. Interviews with voluntary and community sector key informants

The semi-structured interviews with VCS KIs were also guided by an interview schedule (available in supplementary material (1). The schedule included general questions about the remit of the organisation the informant was from and their role within it, the support they provide to families during the perinatal period and how women access their service. The schedule also included questions about specific groups who are known to experience inequalities (as identified in previous research). Informants were also given the opportunity to identify any other groups who may experience inequalities. A range of topics were covered in the interviews relating to barriers and facilitators of identification and management of PMH difficulties, the role of VCS organisations in the perinatal pathway, and challenges due to the COVID-19 pandemic. Informants were also asked to suggest recommendations for reducing inequalities. Interviews were conducted by (CN), lasted between 30 and 60 min and were audio-recorded and transcribed.

### 2.3. Data analysis

#### 2.3.1. Quantitative

Quantitative data was generated through the HCP survey. Response rates and descriptive data on the demographics and job roles of respondents were summarised in order to describe the sample of respondents. These data and responses to closed questions were analysed using basic descriptive statistics including frequency counts and cross-tabulation.

#### 2.3.2. Qualitative

All qualitative data was managed and analysed using NVivo. We adopted the framework method of analysis and followed a series of steps, including data familiarisation, coding, developing and applying an analytical framework, charting the data and finally interpreting the data (16, 17). The analysis was conducted by a team of multidisciplinary researchers (ZD, SB, SM, CE, LP, CN) including those with policy expertise, applied research methods, psychology and health sciences backgrounds. An initial meeting was held to aid the development of a collective familiarity with the data. In advance of the meeting each member of the team read the same two transcripts and made notes about initial impressions. We worked through the transcripts together in the meeting noting and discussing salient points and ideas relating to our research aims and objectives. The two researchers who conducted the interviews were invited to share their reflections. Following this meeting we developed a set of preliminary codes, based on our discussion, guided by the systems approach adopted in our wider project and informed by our own previous research (6, 18, 19) and wider literature (20, 21). Codes comprised barriers and facilitators to PMH care and were coded in relation to the level of the “system” (i.e., individual, family, practitioner, service including service managers and commissioners, government and society) and the point in the PMH pathway (i.e., disclosure/identification, referral, access/uptake) in which they were observed. The coding was applied to the transcripts and a series of coding meetings held to refine the codes and discuss emerging issues.

### 2.4. Synthesising findings and developing recommendations

Once coding was complete, the analysis team met to discuss the organisation of codes into framework categories and the development of an analytical framework. Influenced by previous literature and our own work, the codes naturally organised around a socio-technical framework to capture the complexity of the interactions and multi-directional relationships between (1) processes (e.g., ways of working and actions taken), (2) people (including practitioners, women, and others), (3) technology (including but not limited to the use of information technology); all situated in (4) the wider system (i.e., the wider organisation, here the collection of services caring for perinatal people) (22, 23). While the four main headings were drawn from existing approaches, the categories within them were developed inductively from the data by the authors. In applying a socio-technical framework, we were able to enhance our understanding of how and why inequalities arise and what might be done to alleviate those inequalities. This can be done by identifying possible solutions (areas for action) and how these may help to overcome barriers, with consideration of particular inequalities, together with identifying where these actions need to occur.

We assigned the data to the framework (also known as indexing) and then proceeded to the charting stage of our analysis which involved generating a matrix that summarised all interviews and survey responses by framework categories. Our interpretation of the data was pragmatic and driven by our objective to develop actionable recommendations for local policy and practice.

## 3. Results

### 3.1. Sample characteristics

#### 3.1.1. Healthcare practitioners

The survey was distributed to approximately 1,900 HCPs. Of those, 201 (11%) HCPs clicked on the survey link and 145 completed the survey giving an estimated response rate of 8%. The roles held by the 145 healthcare practitioners who completed the survey varied: 66% (*n* = 96) reported working in universal services (e.g., midwives, health visitors and GPs); 24% (*n* = 34) worked in specialist community or inpatient PMH services; and 10% (*n* = 15) identified themselves as specialists working in universal services (e.g., a PMH specialist midwife working in a community team). IAPT practitioners in the survey self-identified differently: some as working in universal services and others in specialist services).

Table 1 summarises the variety of professions represented in the sample, including midwives or specialist midwives (27%, *n* = 39), health visitors (8%, *n* = 11) and mental health nurses (8%, *n* = 11). Over a third of the participants (36%, *n* = 53) had worked in their current role for 6 or more years. However, the majority of participants reported to have been in their role between 1 and 5 years (53%, *n* = 75). Relatively few respondents reported holding their role for less than 1 year (11%, *n* = 17).

**Table 1:** Distribution of professional roles of healthcare practitioners, survey respondents (*n* = 145).

Profession	*N*	(%)
Midwives	31	21%
Specialist midwives (e.g. mental health role)	8	6%
Health visitors	11	8%
GPs	7	5%
Mental health nurses	11	8%
Community psychiatric nurses	10	7%
Senior perinatal mental health practitioners/ other senior practitioners	10	7%
Psychiatrists/psychologists	9	6%
Wellbeing practitioners/counsellors	10	7%
Cognitive behavioural therapists	8	6%
Other mental health practitioners (liaison/ early intervention psychosis)	5	3%
Support workers (e.g. peer support)	5	3%
Clinical service leads	5	3%
Other nurses	5	3%
Others (detail not provided to preserve anonymity)	7	5%
Not reported	3	2%

The majority of HCPs identified as White British (85%, *n* = 124). The remaining 15% (*n* = 21) included people from a wide range of other ethnic backgrounds or those who preferred not to disclose their ethnicity (*n* = 1). Participants were mostly female (89%, *n* = 129). Twelve per cent (*n* = 17) spoke other languages in addition to English.

#### 3.1.2. Women

**Figure 1:**
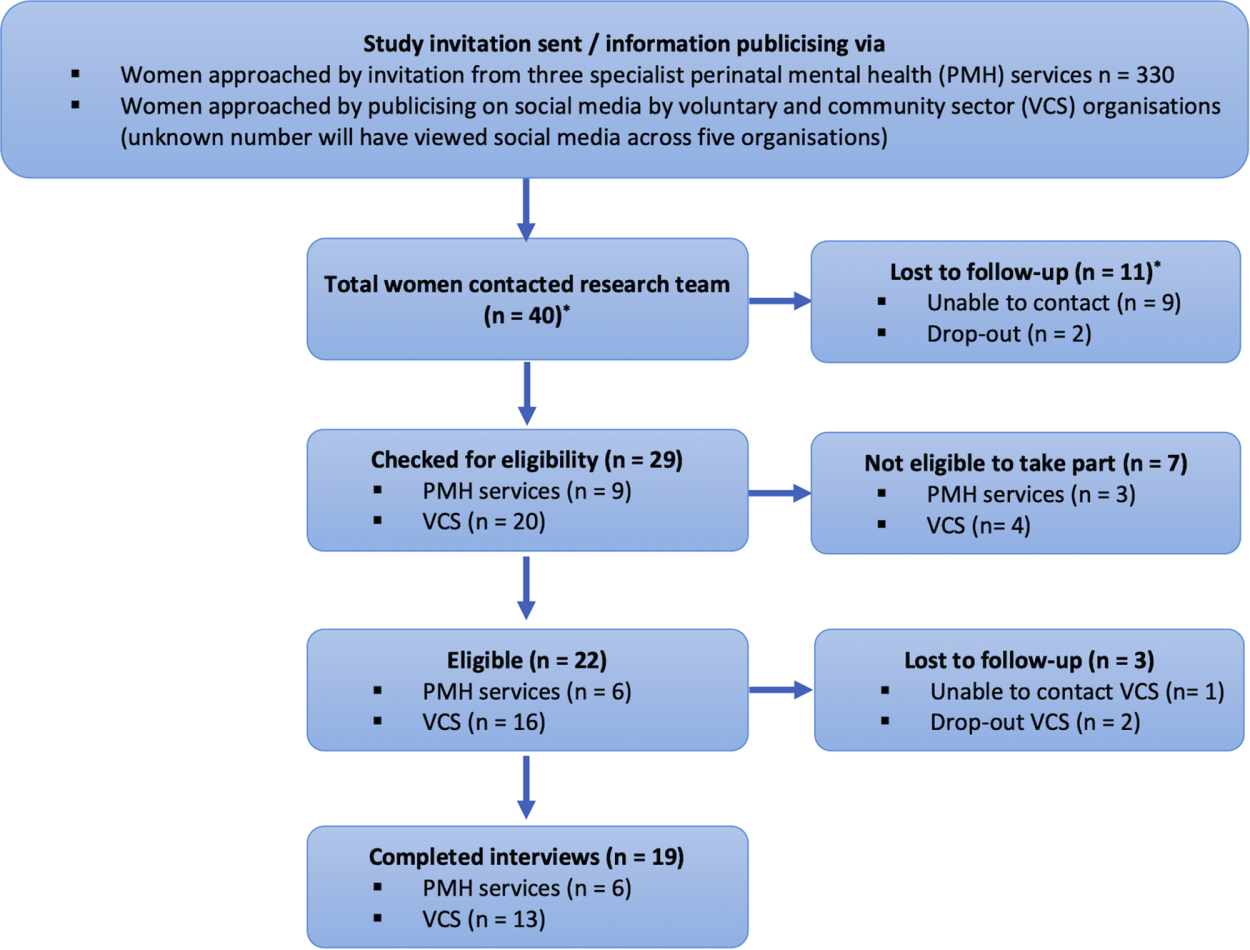
Flow of participants for parent interviews *entry route *via* PMH/VCS unknown before eligibility had been checked.

Figure 1 illustrates the flow of women who were approached and participated. The characteristics of the 19 women who consented to interview are summarised in Table 2.

**Table 2:** Characteristics of sample of women interviewed (*n* = 19).

Characteristic	Description of the sample
Referred to specialist PMH service	6 women were referred to specialist PMH services; all had a mental health diagnosis. Women ranged in support accessed, including crisis team, psychiatrist, perinatal psychologist, intensive support service, PMH services (unspecified).
Onset of difficulties impacting in the index perinatal period, where reported	Antenatal onset = 13
	Postnatal onset = 6 (including some following pregnancy loss in index pregnancy)
Previous or ongoing mental health difficulties/emotional concerns (if mentioned)	14
Type of difficulties impacting in the perinatal period, as expressed by the women	Anxiety, feeling really anxious, nervous, trauma, having panic attacks, flashback, episodes of psychosis, depression, postnatal depression, depressive mood, low mood, bonding problem with children, traumatised by the losses - afraid going to lose child, suicide ideation/ feeling suicidal, tendency to self- harm (e.g. want to take extra medication), being/ feeling angry, don’t feel like doing much, feel lonely, thinking too much, feeling numb, felt overwhelmed, low confidence, not smiling in family pictures - happiness was wiped off my face, stopped going out, stressed, struggling to do simple tasks, really struggling, feeling shut down, sleep problems
Mental health diagnosis, where specified	11 including depression, postnatal depression, anxiety, borderline personality disorder, episodes of psychosis
Perinatal loss (pregnancy loss or baby loss, where mentioned)	10 (including 3 in the index pregnancy)
Currently pregnant	2
Parity (number of previous births, i.e. excluding current pregnancy or any previous early loss)	One = 4, two = 6, three or more = 9
Age	Mean age 35 years (range 24–43 years)
Ethnicity	Pakistani = 9, Bangladeshi = 3, Indian = 2, Other mixed = 2, Other Asian = 2, White British = 1
	Note: country of birth and nationality not routinely asked
Interview language	All women spoke English. 12 women commonly spoke other languages but opted to be interviewed in English. Languages included: Punjabi = 6, Urdu = 3, Bengali = 2, Gujarati = 2, South East Asian language = 1
SES (socioeconomic status)	Mostly low-medium (based on eligibility questions asked)
Currently in a relationship	18

#### 3.1.3. Voluntary and community sector key informants

From our shortlist of 20 VCS organisations, 11 responded in the time frame and identified a total of 12 KIs between them (two KIs worked for the same organisation). The VCS organisations involved in this research worked closely with women and provided a mixture of emotional support (e.g., listening-in, counselling, providing fitness classes and mental wellbeing courses) and practical support (e.g., creche/ stay and play facilities, and provide funding for travel and technology when needed) to women in the perinatal period. VCS services also provide support to women to access statutory services.

### 3.2. Quantitative findings – who experiences inequality?

83 respondents (53 universal HCPs, 30 specialist HCPs) answered questions about the groups experiencing inequalities. The majority (*N* = 70, 84%) reported that they have observed PMH inequalities among at least one of the pre-specified groups (respondents were able to select multiple options – their choices were not constrained). However, 13 (15%) HCPs (5 in universal services and 8 in specialist services) reported that they have not observed any inequalities among the pre-specified groups. Respondents indicated that women who do not speak English were most likely to experience inequalities in the PMH pathway (*n* = 70, 84%), this group was followed by ethnic minority women who speak English (*N* = 56, 67%), those experiencing socioeconomic deprivation (*N* = 55, 66%) and individuals with learning difficulties (*N* = 54 65%). Concerns were also noted for lone women (*N* = 38, 46%) and multiparous women (*N* = 35, 42%). Respondents were given the opportunity to identify any other groups of women they believe experience inequality in PMH care. A wide variety of characteristics were identified, including gender and sexual minority parents (LGBTQ+); young people/teenagers; women with complex needs; women with experience of multiple/past traumas; and women experiencing domestic abuse.

Regarding points in the care pathway (respondents were able to select multiple options – their choices were not constrained), universal HCPs most frequently identified inequalities in disclosure for all groups, followed by identification and referral. For example, women who do not speak English were viewed as most likely to experience inequality relating to disclosure of their mental health difficulties (*N* = 44, 92%), however these women would also be disadvantaged from being identified (*N* = 38, 79%) and subsequently referred (*N* = 25, 52%). Similar patterns were also observed for the low literacy group (disclosure: *N* = 38, 79%; identification: *N* = 24, 62%; referral: *N* = 24, 50%).

Specialist HCPs most frequently identified inequalities in take-up, except for the learning difficulties group for whom HCPs identified fewer inequalities in relation to take-up (*N* = 9, 64%) compared to referral (*N* = 11, 79%) and access (*N* = 10, 71%). Significant concerns were also indicated for all groups about being able to access treatment/services. For example, for women who do not speak English, 73% (*N* = 16) of specialist HCPs reported that these women are likely to be disadvantaged when being referred, 68% (*N* = 15) indicated these women are likely to experience inequality in access and 86% (*N* = 19) indicated inequality in women taking-up the treatment.

### 3.3. Qualitative findings: barriers and facilitators

**Figure 2:**
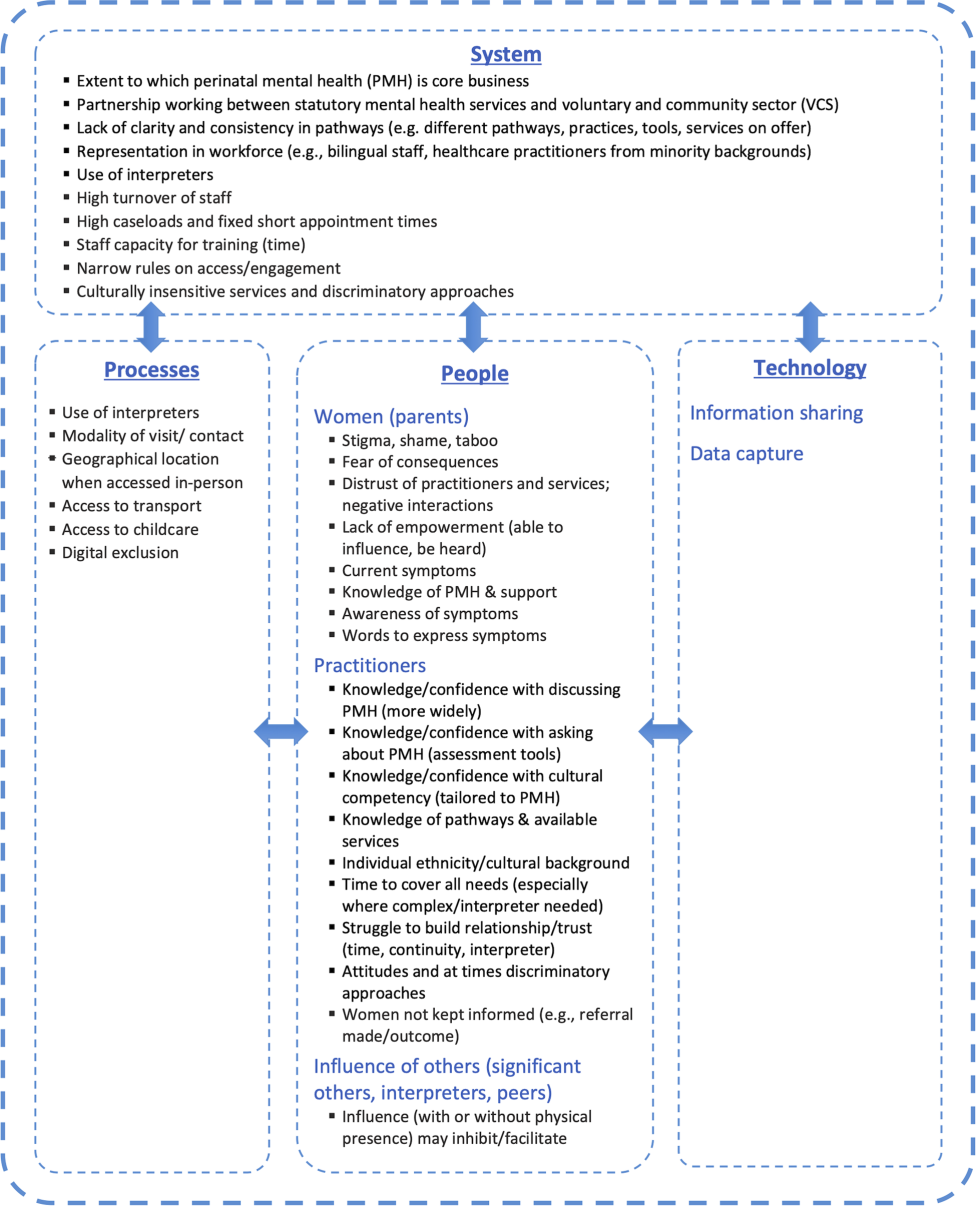
Barriers and facilitators mapped on a socio-technical framework.

Our analysis produced a range of barriers and facilitators, organised according to the different levels (e.g., processes, people, technology and system) within the socio-technical framework, as shown in Figure 2. In some cases these influences operated simultaneously across different levels. Each of the levels and the barriers/facilitators within them are described below, with barriers/facilitators identified using bold text.

#### 3.3.1. Processes

Two key process-related aspects concerned the use of interpreters and the modality of visit/contact, both of which have the potential to deepen inequalities. Appropriate language provision was understood by HCPs and VCS KIs as key and that, despite there being funding available in the NHS for interpreters, provision was inconsistent.

*“One thing which I think they could do is always use interpreters, and that is missing so often. Either from GP appointments, you know, midwifery appointments, maybe even in counselling[…] everyone has a right to be understood, and there is funding for interpreters in the NHS, but frequently they’re not used.” [VCS KI 4]*

*“Deaf people often find it difficult to access services in general. Interpreters can be difficult to access and telephone support is more difficult to arrange.” [HCP 93, universal services, Mental Health Nurse]*

Some indicated that where language barriers impacted on communication, there is pressing need for continuity.

*“Language barriers create difficulties when try to communicate…and support understanding of the client[…]recently I was tasked with trying to recruit this client through use of a phone interpreting service which was very difficult and may have been the reason why the client declined[…]It is more difficult to establish relationships with clients through an interpreter but it can be achieved if they see you regularly enough.” [HCP 78, specialist in universal service, Other Nurse]*

Modality of visit/contact was emphasised particularly where there are language barriers, and it is acknowledged that this has become a more immediate consideration of the COVID-19 pandemic.

*“The limited number of contacts that mums and dads have with professionals is limited and some contacts are telephone only. These are missed opportunities to pick up on women mood as it is unlikely that they will state how they are feeling over a phone.” [HCP 53, universal service, Other Nurse]*

While the importance of in-person contact was voiced - both for conversations including mental wellbeing and as part of socialising for women and babies - it was recognised that remote provision could potentially help to overcome some practical barriers with access. For example, barriers concerning process were the geographical location when accessed in-person (e.g., being in a different city), access to transport and access to childcare:

*“The financial barriers are quite real because when you’re not allowed to work and you’ve got a specific amount of money that is just for your food and clothing because being, when you’re an asylum seeker you just get £5 a day per person and that is for your food, that is for all your toiletries including everything and they just forget about your travel and if I had to go over […] for the mental health face-to-face appointment I had to buy bus tickets or if I was getting late, maybe I had to get to the taxi and it wasn’t just possible.” [Woman 11]*

It was evident from comments across all participant groups that the potential benefits of remote provision needed to be balanced with recognition that remote services may present digital exclusion, both concerning knowledge or confidence with online communication and also, for example, the need for an appropriate device with adequate data or requiring appropriate funds.

*“We’d be working with families, refugees and asylum seekers and they wouldn’t have any devices[…]there was no way for them to engage with our service, or when I was referring families to other services[…]like perinatal mental health service, again they were dependent on families having electronic devices[…] it did feel very, very much like big sort of groups of the community were being excluded from, from that.” [VCS KI 11]*

#### 3.3.2. People

Turning to the level “people” offered a way to understand the barriers/facilitators that operated in different groups. We identified these as relating to women (i.e., parents themselves), practitioners, and the wider influence of others.

##### 3.3.2.1. Women (parents)

Concerns relating to stigma were highlighted both with women and with VCS KI interviews, and specifically identified as likely to be compounded in women from marginalised communities, including ethnic minority communities. These related to discussion of PMH being seen as “taboo” and women experiencing challenging emotions, including shame.

*“in some cultures, mental health is simply not spoken about - which proves difficult for some women to disclose their mental health difficulties and the appropriate referrals being made.” [HCP 41, specialist in universal service, Midwife]*

HCPs suggested that the pandemic may be having a positive impact on public awareness of mental health and that this might support the normalising of help-seeking around PMH for both women and partners and contribute towards reducing stigma.

*[Survey question: Have any of these barriers been compounded or alleviated by the COVID-19 pandemic and why?] “I think women and partners are more able to talk about MH. Everyone recognises it has been a tough time.” [HCP 44, universal service, Midwife]*

The fear of consequences of disclosing PMH needs was repeatedly identified as a barrier. These fears were linked to stigma and included aspects such as judgement and abandonment in communities, fear of being judged by practitioners, fear of unwanted involvement with services (including social care) and for those who were forced migrants with unsettled status, fears of dispersal.

*“I think [they worry] they may be sent to a detention centre and so then the support would stop, or they’ve been dispersed […] they want the help but they don’t know, they’re fearful to trust, it's huge.” [VCS KI 4]*

For some, these fears were compounded by distrust of practitioners and services. HCPs, VCS KIs and women themselves reported that women in marginalised communities were particularly alert to previous negative interactions, experienced either directly or within their communities. Anticipated negative interactions could deter people from confiding in practitioners or using services. Here, VCS voiced this distrust and the work done by their organisations.

*“Disclosure - people are afraid that they will be seen as a “bad mother”[…]Worse in deprived areas, worse for people with learning difficulties or low literacy, as they often don't trust services due to bad previous experiences and fear children will be removed.” [HCP 9, universal service, GP]*

The COVID-19 pandemic added new dimensions to both fear of consequences and distrust of practitioners and services. HCPs and VCS KIs reported anxiety amongst women about contact with health practitioners and services due to risk of transmission of the virus and there was a perceived reduction in help-seeking behaviours and contacts as a result.

*“Women out there, especially in the South Asian or Black communities have been avoiding going into hospital, getting any support from a perinatal perspective because they’re scare that it's going to be used, that this pandemic is an opportunity to be used to kill us off. This is the feeling that we’ve been hearing.” [KI 3]*

Some women felt they had limited influence on their own care or the decisions made about them; expressed here in relation to care more widely.

*“Look at the end of the day, I’m the patient, this is my decision. Whether or not you agree, I still have to consent. And you’re not supposed to be coerced, that's another important thing in medicine, you’re not supposed to be coerced into decisions, and I feel a lot the times I was actually coerced. I was made to feel like if I don't do this, then this will happen. Something negative will happen.” [Woman 1]*

This lack of empowerment was something that VCS spoke of actively trying to tackle.

*“Creating spaces where people can have their voices heard, where they can say what they need and what they want and not necessarily what professionals think are best for them […] it may be that women in certain communities that therapy isn’t the right intervention for them, isn’t what would be most effective, it may be that they need more community groups or they need more peer support, or they need more crisis lines […] it may be that those people need something different or they want something different or they might want more, to feel more empowered in their care and their treatment.” [VCS KI 10]*

Together, all of the above can be understood as the need for emotional safety, as summed up by this KI:

*“All of these particular issues are things that can stop women from feeling safe or confident enough to try and access services.” [VCS KI 7]*

Information, education and knowledge were raised in relation to women's current symptoms (including the extent to which these were interpreted by women as related to PMH), knowledge of PMH and support, awareness of symptoms, and having the words to express symptoms. Such barriers could impact most early in the pathway, concerning disclosure and identification, but also be felt further along in relation, for example, to the provision of appropriate and accessible support. Some participants highlighted the negative impact of the pandemic on awareness of and experience of symptoms.

*“Not having the words to describe how they are feeling in a way which our culture or someone who does not struggle with a learning disability, is able to express. For example, they may focus more on the physical aspect of a problem rather than how they are feeling and therefore a mental health difficulty is missed.” [HCP 74, White British, specialist service, role omitted to prevent identification]*

*“I think [women from South Asian backgrounds] do suffer from it, but they don’t know what it is either. So then, like, obviously when I went to the GP and because you know when you’re speaking you think about ‘you’re tired more than yourself’. That's why I went to the GP […] and then when they were like saying anxiety and stuff and like I’ve never heard about the word in my life. And then they were like oh this and they’re having to explain it. I’m like ‘oh my God! Like I’ve been going through this all my life then.’ It's not just now. It just got worse now because I have to stay home [in the pandemic].” [Woman 5]*

##### 3.3.2.2. Practitioners

Concerns were identified regarding practitioner knowledge and confidence. This included lacking knowledge and confidence in relation to discussing PMH in general and asking about PMH (using assessment tools). Moreover, participants indicated that these were compounded by practitioners lacking cultural competency, including specifically in relation to PMH and varied experiences and presentations.

*“Perinatal mental health and mental health in many communities is a taboo subject, even in the 21st century, and is hidden away and not encouraged to be spoken about. The word depression is not used in many different languages, and it may present itself with somatisation which many professionals are not aware of and go down the route of investigating the physical symptoms rather than being aware that the underlying problem is mental health.” [HCP 53, universal service, Other]*

Practitioners sometimes lacked knowledge of pathways and available services, both generally and including those that may be most appropriate for people from diverse ethnic, faith and cultural backgrounds. In some cases, this was exacerbated by the pandemic, with HCPs and VCS both reporting a lack of information about whether and how services could be accessed remotely or if they were still running at all.

*“Still seems to be issues with midwifes and health visitors not always signposting/referring in and watch and wait is adopted.” [HCP 16, universal service, Cognitive Behavioural Therapist]*

Women identified contrasting perspectives on whether having a shared ethnic/cultural background with a practitioner could enable or deter disclosure, with these examples highlighting the role of religion within culture:

*“the psychiatrist I spoke to was same ethnic background as me, and the same religious background as me […] he said, “ just feel like we should probably try to keep you away from the medication if you can, you know, get yourself together that would be a better idea.” And he then said to me that, you know, “You’re a Muslim at the end of the day and any thoughts of suicide or self-harm, you know, it's not permissible in our religion, in Islam,” […] I appreciated from a religious perspective his advice, just from a medical point of view I was a bit disappointed in that as well, because I almost felt guilty asking for the help, or wanting the help, or even feeling suicidal.” [Woman 4]*.

*“I definitely think it would make a difference…you want that kind of…familiarity rather than people that are not your own culture or your own religion dealing with you.” [Woman 16]*

All groups of participants named challenges concerning having the time to cover all needs. This was raised in relation to (perinatal) mental health in general but identified as particularly challenging where needs were complex (e.g., housing) or an interpreter was needed.

*“Only having 10-minute GP appointments is not enough time to discuss sensitive information about mental health. Someone might want to develop a trusting relationship with someone first, before divulging information about their mental health”. [HCP 74, specialist service, Cognitive Behavioural Psychotherapist]*

This was seen as a contributing element in the struggle to build relationships/trust, which was impacted by lack of time, lack of continuity and potentially the use (or absence) of an interpreter. There were however examples where people shared positive experiences about relationships with practitioners:

*“She [midwife] knew what was happening, she knew what she was saying, she was really like empathetic to my needs. There was no rushing, she was really, really good. […] She would just like listen, she would understand, she knew the area, she knew like families and things […] But the fact that she just listened and she didn’t judge, that was really, really helpful.” [Woman 13]*

VCS KIs emphasised the importance of building such trusting relationships and several felt that the way that the services operated helped to enable them to work in ways that promoted these, which they contrasted with arrangements in the NHS. Of concern, there were also examples where attitudes and at times discriminatory approaches where shared. These linked to wider distrust of practitioners and services (named previously in relation to people: women) and had potential to present barriers to PMH access:

*“You know, whenever you’re an ethnic minority, a woman, and a Muslim, you’ve got three strikes against you […] you feel like you have to prove yourself so much more than other people […] just the figures and statistics show Black and Asian women are more likely to die in childbirth, they’re more likely to have miscarriages. Why is that, you know, there has to be a reason for that? […] Like perhaps it's the case that, you know, the White medical staff are making assumptions about these women and they’re not really taking their concerns seriously”. [Woman 1]*

Lastly, a barrier/facilitator expressed in relation to practitioners concerned the extent to which women were kept informed; this included in relation to referrals being made and the outcomes of referrals.

*“Once they felt I was okay to be discharged they just discharged me […] But after that there's been no follow-up from my GP, from my midwife, or any of the services, I haven’t heard anything really. I got a letter in the post saying that I was under the care of some postnatal team, I have absolutely no idea who that team was, they never introduced themselves to me, I never got any contact or anything at all.” [Woman 4]*

##### 3.3.2.3. Influence of others (significant others, interpreters, peers)

The influence of others related to three broad groups: significant others (i.e., partners and other family members or close friends), interpreters and peers. There were contrasting perspectives on the presence of partners and other family members in mental health assessment and the potential to both facilitate and inhibit conversation. It was also apparent that influence of others can be felt without physical presence (i.e., anticipated reactions of others). For example, there can be concerns about bringing shame or other negative judgment to a family through sharing what may be considered “private” or “family” concerns. This may also extend to the influence of wider communities.

*“Even when a woman has disclosed and the referral is made, due to often living in multi-generational households, it is not always easy for women to have private telephone conversations with mental health services. Because of this, referrals are often declined by women.” [HCP 41, specialist in universal service, Specialist Midwife]*

Flexibility in relation to physical presence of partners and other family members was limited during the pandemic lockdowns. Some practitioners felt this meant they were able to have more open discussions about PMH, whereas others were concerned about partners feeling excluded (with increased risk of their own PMH difficulties not being spotted). Women reported that the absence of significant others in appointments during this time had a detrimental impact on women's mental health.

*[Survey question: have any of these barriers been compounded or alleviated by the COVID-19 pandemic and why?] “Due to partners/friends and family not being able to attend the wards during the pandemic I believe it has helped us be able to ask the right questions when the environment feels safe and secure. Women do not feel pressured and are able to speak openly without others knowing.” [HCP 103, universal service, Midwife]*

*“I think, ever since COVID has happened I think it's made people realise that having somebody with you as a family member, either be your husband or your sibling, or your mum, or anybody who you know, is so valuable to your mental health.” [Woman 17]*

Interpreters are discussed in relation to *processes* but also apply to *people*, here linking with the extent to which interpreters and women may be known to each other within local communities. In this example, although not specific to interpreters, potential concerns about “community links” were identified:

*[Survey question: Example of good practice for ethnic minority women who speak English] “It is not as might be expected. I have had some BAME women decline contact with BAME staff simply because they think that their confidence may be broken due to community links. However contact was made with white staff. Nevertheless, BAME staff have quickly made links where white staff failed previously.” [HCP survey 19, specialist service, Psychiatrist/Psychologist]*

Another influence that repeatedly featured, particularly in VCS KI interviews, was the influence of those working within VCS organisations who may be perceived as peers. This includes the potential for peers to positively enable conversations about PMH, including disclosure and onward help-seeking or taking up the offer of support.

*“It was really good honestly. I kind of miss that now. I was basically with them for a year, like we’d just talk about everything and like you just know you’re not alone anymore. Like you’re not the only one that's going through it. It's really amazing. Like I couldn’t have done it without them because I would just like tell them anything and everything.” [Woman 5]*

*“One of the things they talk about in the group is, you know, how great it is, you know, when one person talks about their mental health issues.” [VCS KI 7]*

#### 3.3.3. Technology

Only two technological barriers were identified by stakeholders. One related to instances of poor information sharing with women and between services.

*“[in response to a question about how to tackle PMH inequalities] Multidisciplinary team working sharing all information and being able to access all information required for example uploading to all systems of different ones are used” [HCP 46, universal service, midwife]*

*“It was quite stressful because obviously they all spoke to each other anyway, and I had to keep repeating myself like the same thing to each person. I was like you already know all this, you all speak to each other […] I was just saying the same thing over and over again which made me stress out even more.” [Woman 2]*

*“The only thing that I had an issue with was the very first appointment, where I was questioned as to my number of children. And I did say to her straight away that because of my previous pregnancies there should be a little teardrop sign, eye on my system, on my file, which is what I’ve been told previously, that they should be on there” [Woman 13]*

There were examples where information sharing had been further compromised in the context of the pandemic. As noted above in the context of practitioners, an example of this was women not consistently being kept informed about the outcome of their referrals; here, identifying the role of technology.

The other key barrier indicated in relation to technology was data capture where HCPs gave examples in relation to the lack of flags or markers for particular characteristics in recording systems as well the need for better data to be captured and aggregated.

*“never seen any identification on medical records of low literacy/learning difficulty etc flagging this up to professionals” [HCP 29, universal service, other mental health practitioner]*

*“[in response to a question about how to tackle PMH inequalities] Collecting good data on different groups” [HCP 14, specialist service, Senior PMH Practitioners]*

Although HCP references to data capture were not detailed, it was also evident in establishing mechanisms for participant recruitment for this study that services do not consistently hold complete or accurate data concerning background characteristics and information relating to engagement with the service is not readily accessible. Without this, it is impossible to fully understand where inequalities exist or monitor measures to address them.

#### 3.3.4. System

System-level barriers were evident, particularly in relation to the extent to which PMH is core business. This was identified as impacting directly on aspects at the *people: practitioner* level. For example, some expressed that there is a focus on physical health that is connected with there not being adequate time to fully address mental health concerns.

*“So, if they didn’t rush it as much and I could actually, you know, talk to them and tell them how I felt and stuff, if they focused on the mental health as well aspect of that then that would be much better.” [Woman 15]*

Similarly, PMH not being seen as core business was linked to aspects such as lacking adequate knowledge and confidence *(people: practitioners)*. This appeared connected to (at times, limited) partnership working between NHS services and VCS organisations. For example, partnership working was identified as being a way to facilitate knowledge-sharing in relation to PMH, its assessment or pathways and available services *(people: practitioners)* and addressing cultural competency *(people: practitioners)*, to in turn buildemotional safety *(people: women (parents))* and address distrust of practitioners and services *(people: women (parents))*.

*“[in response to a question about how to tackle PMH inequalities] Using link workers within community to build relationships with women alongside the community midwife and enable them to communicate their thoughts, concerns. This would enable the woman to also understand what services are available and what they can be offered.” [HCP 28, specialist in universal, Specialist Midwife]*

*“Better communication between midwifery services and primary care and vice versa - midwives used to work in the surgeries and we had excellent links but this has been eroded over the years by moving them out of primary care. This means we no longer know our midwife teams and vice versa. In the past we would have had a chat about patients and discussed best way forward and now sometimes feels like passed from pillar to post.” [HCP 8, universal service, GP]*

*“In terms of perinatal mental health specific services we don't provide counselling or anything like that but what we do is we signpost and work with other local organisations […] if the NHS work with organisations like us they ensure that our staff are trained, they ensure there is some sort of funding in place for that and funding in place to keep organisations like us going then that kind of helps. So if there is that joined-up thinking right from the ground upwards then it means that like I say that kind of filtration system is there.” [VCS KI 9]*

Lack of clarity and consistency in referral pathways (e.g., different pathways, practices, tools, services on offer across different geographical areas) was described as a barrier. VCS KIs noted that while they are well placed to build relationships and create safe environments that enable women to disclose their mental health difficulties, lack of clarity about referral pathways and available services acts as a block to those women accessing treatment or specialist support.

*“We have regular conversations about this, it is so hard to access mental health services for women. It's hard and it's frustrating and it's dispiriting […] For the women who are experiencing mental health issues, who are trying to get their head round what services are available and how they access them, I’ve got no idea because we feel like banging our head against a brick wall and we do it all the time […] there's no consistency and that makes it really hard to support women accessing mental health support.” [VCS KI 7]*

Comments about the potential for shared ethnic/cultural background of staff *(people: practitioners)* to be a barrier/facilitator were greatly varied but one of the most frequently discussed aspects. This linked at the system-level to a lack of representation in workforce (e.g., bilingual staff, minority HCPs). This lack of diversity could be seen as contributing to aspects such as lacking cultural competency *(people: practitioners)* and distrust of practitioners and services (people: women (parents)).

*“Having a diversity inclusion worker speaking directly with service users of all backgrounds and asking for their help and feedback in order to develop services” [HCP 60, specialist service, Senior PMH Practitioners/Other Senior Practitioners]*

*“Services should reflect the populations that they serve. We need staff from the same cultural background as the people who need to access our service. Too many white staff.” [HCP 19, specialist service, Psychiatrists/Psychologist]*

The use of interpreters was relevant at the *system* level as well as in relation to *processes*. This included not only the impact for women but also the need for longer appointment times. In addition, there were concerns about the extent to which interpreters may need specialist knowledge and training to work effectively in PMH.

*“Interpreting service - takes more time to use and therefore cuts down the time the professionals have to support clients within the session. Meanings may be lost and understanding is difficult to get across as it is unclear if the interpreter fully understands what the [support] is about. Uptake of interpreters means professionals always have a different one for any given session. It would be helpful to have specific interpreters allocated to specific services so that they gain an understanding of the work being carried out and therefore support client understanding better.” [HCP 78, specialist in universal, Other Nurse]*

Concerns about time to cover all needs *(people: practitioners)* were linked to wider challenges at the system level, concerning high turnover of staff, high caseloads and fixed short appointment times, and wait times. This was identified as having implications for aspects such as building emotional safety *(people: women (parents))*.

*“Limited time during appointments or on the ward. Low staffing meaning only basic care can be provided.” [HCP 46, universal, Midwife]*.

*“What we know is services like the Mental Health Wellbeing service, you know, it's great that we’ve got that but the wait is really-really long for women […] when somebody gets to the point where either they or somebody has acknowledged their kind of mental health needs, what they don’t need is somebody then saying, brilliant, we’ll put a referral in, somebody will get in contact with you in 3 or 4 months’ time.” [VCS KI 7]*

Women, VCS KIs and HCPs reported that the pandemic placed significant additional pressures on visit times and caseloads (linked to short staffing).

*[Survey question: have any of these barriers been compounded or alleviated by the COVID-19 pandemic and why?] “There has been a significant reduction in staffing levels, which has caused our service to cease all antenatal contact unless there are known safeguarding concerns. This therefore significantly increases the possibility of mental health issues not being identified, and women not feeling comfortable enough in the therapeutic relationship to do so.” [HCP 31, universal service, health visitor]*

*“It was because of COVID […] at that time it was all, what's the word, we had to get in and out quickly […] it was very quick and it wasn’t something that you, you can’t just talk about everything, you know, how I’m feeling in those ten minute sessions.” [Woman 15]*

It was noted that staff capacity for training is also relevant for addressing PMH. This concerns both having the time to attend and the time to reflect on and action the learning, for this to be meaningful and effective at creating change:

*“Like every individual practitioner examining their privilege in the way they use power, and their assumptions and their biases like, it's like a massive piece of work for everybody to do and if institutions are going to say that they’re going to do that work then they need to give those practitioners time, support, resources, and it's like real human difficult painful work and it's just, ‘oh do a piece of training’, and you’ve worked it out.” [VCS KI 10]*

Particularly vocalised by VCS KIs was the ways in which narrow rules on access/engagement within the NHS could present barriers to accessing support, particularly for individuals from ethnic minority groups and those living with trauma. In having these rules about access it was indicated that this would also have implications for aspects such as building emotional safety *(people: women (parents))*.

*“I think there has to be more flexibility about appointments […] there really is, you know, a 3 strikes and you’re out type thing that I see so often in services […] But actually […] the very fact that somebody's not able to come to your appointment when you have said you want them at the place that you have said you want them, kind of suggests that they’ve got mental health or there may be other things that are going on […] if we don’t work in a trauma informed way our services are not accessible.” [VCS KI 7]*

*“We try and address the kind of barriers as much as possible but I think in terms of NHS services it's almost, it's difficult because it seems really formal and there's nothing you can do about that. There's nothing the NHS can do about that because it has to be a formal situation that can sometimes scare people off, but I think the more the NHS or, you know, [specialist PMH service] or whichever department, perinatal mental health or wherever, the more they work with community organisations like us who build up that trust and they see that yeah, we're working with the NHS. […] the more they see that, the more that will build up confidence [in NHS services], and quite a lot of these things stem from a lack of confidence.” [VCS KI 9]*

The final barrier identified at the system level was culturally insensitive services, which includes some examples indicative of discrimination.

*“Stop sending opt-in letters, they are an enormous barrier for marginalised groups. They mean that the most pro-active patients with best literacy and best ability to seek help end up using all the resources and getting a better service, and those who struggle to read, struggle to accept that they have a problem, or don't speak much English, or don't like making phone calls simply don't get any access to secondary care MH services.” [HCP 9, universal service, GP]*

*“It was only a couple of times, maybe […] sometimes, you know, before I spoke, you know, they did sort of act as though I was, I couldn’t, maybe they just assumed I couldn't speak English and they were just kind of speaking very loudly…it's a bit patronising. And I don’t know, I’ve not really seen them do that with White ladies.” [Woman 1]*

### 3.4. Triangulating findings and developing recommendations

**Figure 3:**
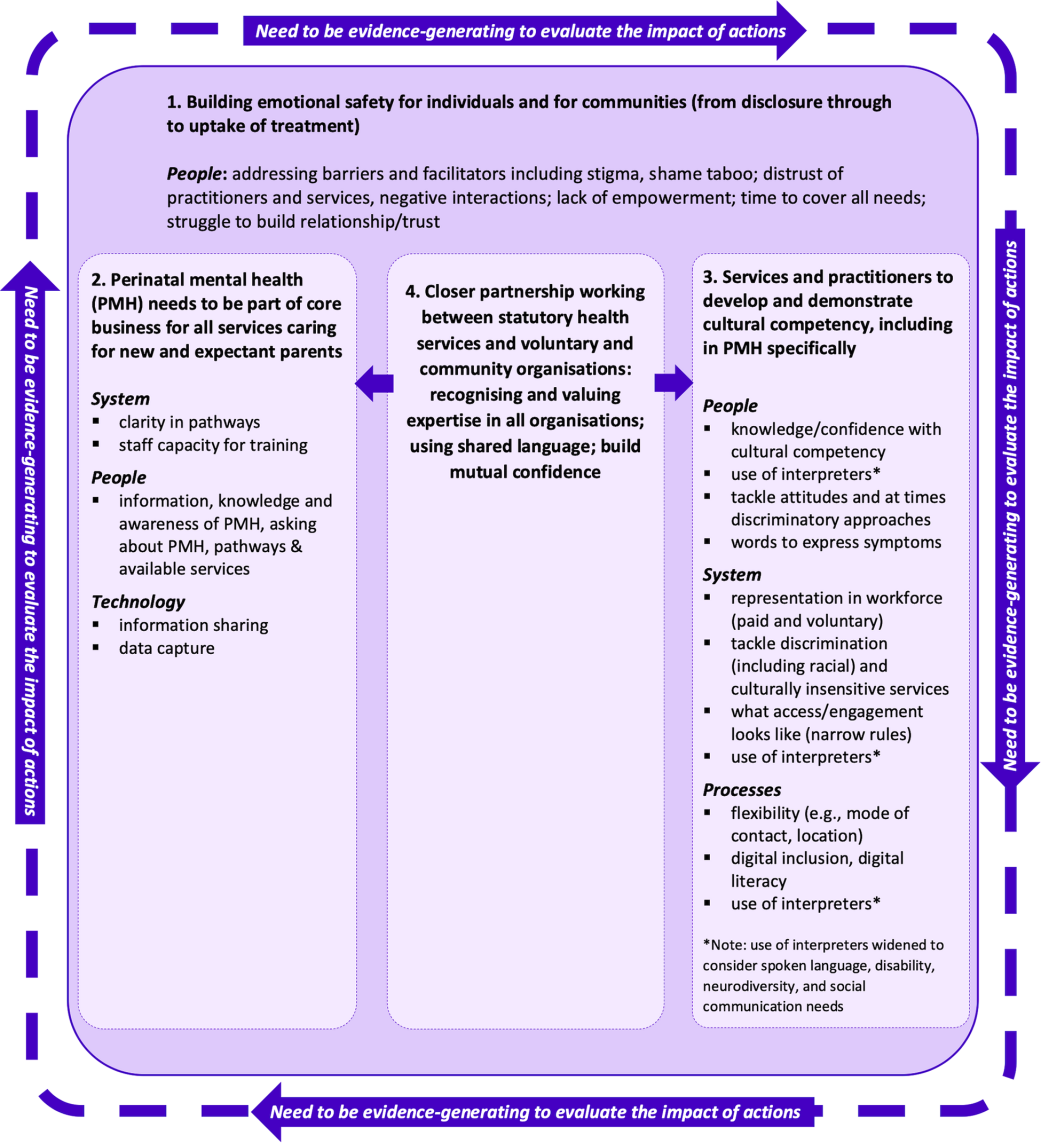
Recommendations for changes at the system-level to tackle in equalities in accessing perinatal mental health care, with named examples of which barriers and facilitators may be addressed by these actions.

Our findings have highlighted barriers and facilitators for women with avoidable and unfair inequalities at numerous levels (e.g., process, people, technology and system), with many barriers and facilitators operating across multiple levels. The complexity and inter-connectedness of our findings point to the need for change at the system-level. To address this, four recommendations were developed from our findings. These are depicted in Figure 3, together with naming example barriers and facilitators that they may address.

Firstly, emotional safety needs to be built for individuals and for communities. Without addressing emotional safety, barriers remain in all steps of the pathway (from disclosure through to uptake of treatment) and contribute to inequalities in care. Building emotional safety involves tackling distrust in services and ensuring that communication needs are met and this may require additional time in appointments. Secondly, PMH needs to be part of core business for all services caring for new and expectant parents. This includes being core business for practitioners and that this is reflected in technological aspects and processes. Thirdly, services and practitioners need to develop and demonstrate cultural competency, and for this to be in PMH specifically. Fourthly, all of these are underpinned by closer partnership working between voluntary sector and statutory health services where expertise in all services is valued, and nurtures shared language and understanding. Although not articulated by stakeholders, we propose too that services need to be evidence-generating to evaluate the impact of any measures adopted to tackle inequalities, including through actively involving marginalised communities as those most likely to be affected.

## 4. Discussion

Barriers and facilitators to accessing perinatal mental health (PMH) care have been the subject of many primary studies and several evidence syntheses (10, 11, 20), and it is unsurprising that similar aspects are identified here in this equity-focused study. This study extends the literature in two key ways. Firstly, we have focused on the barriers and facilitators that cause avoidable and unfair inequalities in PMH care. By applying the socio-technical framework, we foreground a systems approach to better understand avoidable and unfair inequalities in the identification and management of PMH difficulties across care pathways. This indicates the need for action in the wider system (including statutory health services and the voluntary and community sector, VCS) to enable change, with key recommendations presented in our interpretation. Secondly, this study identifies the variable ways in which the COVID-19 pandemic and accompanying rapid changes to ways of working, impacts on existing inequalities. Barriers and facilitators acting on inequalities have seen new dimensions (e.g., to fear of consequences and distrust of practitioners and services) or become more immediate (e.g., mode of contact and digital exclusion). Here we situate these findings in the wider literature before considering the study's strengths and limitations.

### 4.1. The need for system level change

The barriers and facilitators that were evident in the current study resonate with the findings of a recent systematic review of the international literature (11) which examined barriers and facilitators to implementing PMH care across the pathway (i.e., assessment, care, referral and treatment), identifying factors that were framed as related to the individual, healthcare practitioner, interpersonal, organisations, political and societal. Whilst the need for system-level change is articulated, it is not foregrounded and a key difference here is that we focus on inequalities.

Application of the socio-technical framework illustrates that action is needed at the system-level to enable changes in the other levels, i.e., processes (e.g., how care is provided), people (e.g., women (parents), practitioners, and others) and technology (e.g., data capture) to tackle avoidable and unfair differences. This is consistent with the construct of candidacy, i.e., that people's access to healthcare is determined between themselves and health services, shaped by influences and many levels, including how services are configured and how resources are allocated (24). This fits too with the current shift in focus within public health to tackle inequalities in health at the system-level. The systems approach conceptualises individual circumstances and services as events that occur within the wider complex system (25, 26). As we have described here, there are multiple complex causes of inequalities that increase the likelihood of a woman being unable to disclose, have her concerns identified or access and continue to use the right support. It would not be realistic to assume that intervening at the process or person level would be enough to resolve such a complex problem. This approach also has the advantage of moving fault away from individuals (i.e., people) instead locating fault and the need for action elsewhere in the system (27, 28).

It should be noted that this does not minimise individual accountability of practitioners (29) or detract from the need to tackle discrimination.

### 4.2. Building emotional safety

We argue here that building emotional safety is a pre-requisite for addressing inequalities in PMH care. It is increasingly recognised that perinatal services not only need to respond to distress (i.e., through appropriate recognition and management) but also address their own role in contributing to distress. For example, in the context of childbirth-related post-traumatic stress, poor care provider interactions are heavily implicated, however these cannot be separated from the problematic working environments which limit midwives' ability to provide optimal care (30). In the NHS, there is now a good practice guide for trauma-informed care in maternity services and mental health services that may be involved in the perinatal period (31) and this identifies the need for organisational change and transformation of the work culture, so that the environment is experienced as safe, by those receiving and by those providing care (32). Being both trauma-informed and culturally competent is compatible with recognising that people may use services in different ways and that current narrow rules on engagement can deepen inequalities, with change needed in the system rather than the individual who has been offered a service (33).

In this study, we identify the salience of distrust in services for people from marginalised backgrounds and furthermore that this is shaped not only by individual experiences but also anticipated experiences which are shaped by experiences within communities. A novel contribution is that we propose building on principles of trauma-informed care and extending to consider the ways in which marginalised *communities* may be traumatised by services. For this to be achieved, investment is needed across all services that have implications for PMH. For example, in the UK, while there has been rapid investment in specialist PMH services with vast increase in the number of people expected to be seen by these services, this has not been accompanied by investment in universal services that are expected to be key referral routes in. Nor has it been designed with the need to tackle inequalities in access.

The need for emotional safety is heightened in the context of the pandemic. Being trauma-informed emphasises the importance of psychological safety, choice and control (31). Perinatal research conducted in the pandemic illustrates that the pandemic has been a time where people's psychological safety, choice and control have been diminished (34).

### 4.3. Perinatal mental health as part of core business

We argue that building emotional safety requires PMH to be seen as part of core business. It has repeatedly been argued that maternity services need to place equal importance on psychological and physical health (30). However, despite policy developments concerning identification and management of PMH, further change is needed to make every contact count from a mental health perspective (35), i.e., to make every interaction meaningful and seize every opportunity to identify PMH difficulties (36).

For different ways of working to be implemented and sustained, they need to viewed as “core business and priorities” and to not conflict with other priorities (37). This requires systemic change that includes training, supervision and resources which is realistic and transparent about current workforce challenges. For example, providing adequate time in appointments and having staff capacity for training may not be feasible in current systems or without being seen to compete with other priorities. Through application of the socio-technical framework we argue too that technology is a fundamental part of ensuring PMH is part of core business. For example, providing appropriate data capture mechanisms (within the limits of ethical considerations for privacy) is essential for ensuring appropriate referrals. This is necessary as part of effective follow-up and a joined-up perinatal care system (4). Additionally, the existence of such mechanisms themselves also communicate the central importance of PMH. Improvement to data capture systems would also provide infrastructure for accurate monitoring of the service provided and any inequalities that may feature.

Applying this shift in core business to the pandemic, services have seen rapid changes to ways of working due to a shift in priorities. Changes such as moving to remote delivery of services may create additional barriers including those linked to digital literacy and to availability of devices or internet data. However, they can also bring opportunities for more flexible working, for example offering greater choice such as in-person or remote consultation. These examples also demonstrate the ability for services to achieve rapid change in a short space of time. However, in the pandemic the emphasis on infection control appears to have been prioritised at the cost of potentially increasing risk of harm in other ways; for example given the impact of visiting restrictions, emerging evidence about increased prevalence of PMH difficulties including birth trauma, anxiety and depression (38–40).

Having PMH as core business requires culture change and for this to be within all services and individuals that may be involved with PMH, not only those where PMH is the area of focus. This was demonstrated in the current study by views expressed by a diverse range of practitioners. Being trauma-informed recognises the role of all staff in promoting feelings of safety and security, including for example reception staff (31). This study specifically identified interpreters as a key workforce in need of specific training for working with PMH, in addition to highlighting the importance of attending to interpreters' own needs including supervision. Cultural competency is similarly relevant for all staff.

### 4.4. Cultural competency

The need for cultural competency has been repeatedly articulated in wider healthcare literature as a way to address inequalities in healthcare through improving cultural knowledge, skills and attitudinal responses (41, 42). Further, a recent systematic review of ethnic minority women's experiences of PMH conditions and services in Europe identified the need for cultural competency training, noting the variation in provision (3). The urgent relevance of cultural competency for perinatal services is indicated by various findings, including evidence of institutional/systemic racism and discrimination (43–45) and ethnic/racial disparities in the mortality and morbidity rates of mothers and their babies (46) together with evidence of aspects such as different rates of coercion and non-consensual procedures by racial and ethnic identity (47). We propose here an additional need for training specifically about cultural competence *in* PMH given the specific challenges that may present. For example, beliefs about PMH can be highly variable in different sociocultural contexts, which can be closely connected with the powerful influence of stigma (48). Beliefs about PMH also tap into assumptions relating to wider aspects, including parental roles, couple relationships and expectations of what families may look like. For example, professionals in specialist PMH services in the UK work within a competency framework (49) which includes a core competency called the “Perinatal Frame of Mind” that includes the ability to consider mental health and wellbeing in the context of the mother's and partner's race, culture and other protected characteristics. This is relevant for anyone working with women affected by PMH difficulties and yet does not form part of training for those within universal services. This may benefit from including activities to promote continual self-reflection, moving beyond cultural competence to embrace cultural humility (50), as part of addressing power imbalance and may include aspects such as questioning services' reliance on mental health assessment tools that rely on and privilege Western terminologies. VCS organisations are well-placed to contribute to such training, including with input to address continuing ableism within health services.

### 4.5. Partnership working

Through hearing from multiple stakeholder groups, including practitioners within universal and specialist health services and KIs within VCSs, this study suggests that in order to achieve PMH as part of core business and achieve cultural competency, partnership working needs to be valued, together with the expertise offered in all services. This resonates with the position that tackling societal and structural racism and discrimination in maternal health can only be achieved through implementing strategies in partnership with local communities (43).

The need for partnership working has been highlighted by the pandemic, where women may use VCSs differently; for example through finding health services more restrictive and turning to a wider range of information. There were examples of VCSs responding flexibly for example through providing opportunities for connection and support through WhatsApp groups. Similarly, ways of working have changed for meetings between staff, with many meetings now being held online. Here practical actionable recommendations can be offered that may help to nurture partnership working for example joining multi-agency meetings online which may not only reduce practical barriers to taking part (e.g., fewer costs relating to time and travel to attend, and to venue hire) but may potentially also shift power dynamics through not being held in a particular premises. Conversely, it is possible that meeting online may not be as enabling of relationship-building.

### 4.6. Perinatal mental health inequalities in the pandemic

Findings in this study cohere with research pre-dating the COVID-19 pandemic which finds that pandemics reinforce existing inequalities (51) and research within the COVID-19 pandemic, finding that inequalities faced by minoritised ethnic families in relation to universal perinatal services have deepened (15, 43). This is concerning because system issues may not only deepen inequalities in *accessing* care but also directly contribute to vulnerability in the immediate perinatal period (i.e., prevalence/onset), and also carry implications for subsequent perinatal periods (concerning both prevalence/onset and access). Already concerns exist about the rapid growth of mental health difficulties during the pandemic (52, 53), including specifically within the perinatal population (39); and our study indicates the urgent need to foreground inequalities in addressing this. The rapid changes to ways of working within services showcase both the challenges and opportunities created by the pandemic and suggest there may be greater agility than would perhaps have been anticipated. Currently however these changes have not placed PMH as a priority.

### 4.7. Strengths and limitations

Strengths of this study include the involvement of multiple stakeholders groups and inclusion of an ethnically diverse group of parent participants in understanding inequalities. Involvement of multiple stakeholders, together with complimentary work packages (not detailed here) concerning data and the application of the socio-technical framework enabled us to consider influences (such as data capture) which have been relatively neglected in the PMH literature. Due to focusing on particular inequalities in setting the eligibility criteria for parent participation, our learning is more limited concerning other inequalities. As identified in the survey with practitioners and, to a lesser extent, the interviews with VCS KIs, other groups also warrant attention and these include parents with additional needs (e.g., disabilities, neurodiversity, long-term conditions) and parents from other marginalised backgrounds (e.g., LGBTQ + parents); this could be used to test the application of the socio-technical framework and our recommendations more widely. Unfortunately, despite efforts to promote inclusivity, including provision of the study information in multiple spoken languages, provision of interviews being conducted in multiple spoken languages, and publicising through a range of organisations, no parents volunteered who required interpreter support. We also did not hear from any parents who had begun accessing specialist PMH services and discontinued use; although a recent study examining service data suggests that the key challenge may be access rather than utilisation (33). Nonetheless, we recognise that self-selection bias is relevant here, both concerning parent participants and healthcare professionals, given the survey's low response rate in universal services.

We recognise too that the study is limited by recall bias: it is evident that some women struggled to recall details accurately (perhaps due to length of time or due to their health and symptoms at the time) and some women themselves expressed this challenge with recall.

This study is limited to one region in England and we recognise that there are considerable contextual difference within and between countries, both in relation to what PMH care looked like pre-pandemic and the impact of the pandemic on perinatal services more widely. For example, we cannot underestimate that in LMICs, aspects such as COVID-19 disrupting supply chains have had devastating impact for maternal and newborn health (54). We additionally note that healthcare is free at the point of use in the UK; where health systems differ in this regard there will likely be considerably different implications for inequalities in access. Involving service planners and commissioners in future research is recommended.

### 4.8. Conclusion

The stakeholders in our study identified a range of inequalities in PMH identification and management, with some women facing significant disadvantage during the perinatal period which has been further compounded by the COVID-19 pandemic. Our study lends further support to recent calls for a systems approach to tackling inequalities in healthcare. We have outlined a series of recommendations intended to be helpful in addressing avoidable and unfair inequalities across the PMH care pathway. We urge those who are designing solutions and innovating in this area to evaluate the impact of their efforts to build a comprehensive evidence base for reducing inequalities in perinatal mental health.

## Data Availability

The datasets presented in this article are not readily available because this could compromise participants’ privacy. The corresponding authors can be contacted with questions regarding the dataset. Requests to access the datasets should be directed to Zoe Darwin, *z.darwin@hud.ac.uk*; Sarah Blower, *sarah.blower@york.ac.uk*.
